# Associations Between Serum Gut-Derived Tryptophan Metabolites and Cardiovascular Health Markers in Adolescents with Obesity

**DOI:** 10.3390/nu17152430

**Published:** 2025-07-25

**Authors:** Jeny E. Rivera, Renny Lan, Mario G. Ferruzzi, Elisabet Børsheim, Emir Tas, Eva C. Diaz

**Affiliations:** 1 Arkansas Children’s Nutrition Center, Little Rock, AR 72202, USA; jerivera@uams.edu (J.E.R.); slan@uams.edu (R.L.); mferruzzi@uams.edu (M.G.F.); eborsheim@uams.edu (E.B.); 2 Arkansas Children’s Research Institute, Little Rock, AR 72202, USA; 3 Department of Pediatrics, University of Arkansas for Medical Sciences, Little Rock, AR 72205, USA; 4 Departments of Pediatrics, College of Medicine, University of Pittsburgh, Pittsburgh, PA 15224, USA; tase2@upmc.edu

**Keywords:** obesity, tryptophan, gut microbiome, vascular health, flow-mediated dilation

## Abstract

**Background/Objectives:** Gut-derived tryptophan (Trp) metabolites play important roles in metabolic and cardiovascular regulation. Although animal studies suggest their protective effects against metabolic dysfunction, data in adolescents, particularly those with obesity, remain limited. The objective of this study was to evaluate associations between circulating gut-derived Trp metabolites and markers of cardiometabolic, vascular, and platelet health in adolescents with obesity. **Methods:** Data were analyzed from 28 adolescents (ages 13–18; mean BMI = 36 ± 6.4 kg/m^2^). Fasting blood was collected to assess lipid profiles using a clinical analyzer and insulin resistance using the homeostatic model assessment for insulin resistance (HOMA-IR). Gut-derived Trp metabolites were measured by UPLC–mass spectrometry, peak oxygen uptake (VO_2 peak_) by gas exchange during an incremental cycle ergometer test, and body composition by dual-energy X-ray absorptiometry. Platelet spare respiratory capacity (SRC), endothelial function, and liver fat were measured using high-resolution respirometry, flow-mediated dilation (FMD) of the brachial artery, and magnetic resonance imaging respectively. **Results:** Indole-3-propionic acid was inversely associated with diastolic blood pressure (rho = −0.39, *p* = 0.047), total cholesterol (rho = −0.55, *p* = 0.002), and LDL-C (rho = −0.57, *p* = 0.0014), independent of sex and obesity severity. Indoxyl sulfate was positively correlated with fasting glucose (rho = 0.47, *p* = 0.012), and adolescents with impaired fasting glucose had 1.6-fold higher IS levels. Indole-3-acetaldehyde declined with age (rho = −0.50, *p* = 0.007), and Indole-3-acetic acid and indole were higher in Hispanics vs. non-Hispanics. No significant associations were observed between Trp metabolites and FMD, VO_2_ peak, or SRC. **Conclusions:** Gut-derived Trp metabolites, particularly indole-3-propionic and indoxyl sulfate, are associated with markers of cardiometabolic risk in adolescents with obesity. These findings support their potential relevance in early-onset cardiovascular disease risk.

## 1. Introduction

Atherosclerosis is responsible for two-thirds of all cardiovascular disease (CVD)-related deaths worldwide [1]. Evidence from autopsy studies indicates that atherosclerosis begins early in life, with fatty streaks identified in the coronary arteries of children as young as 1 to 9 years old [2]. This early onset of vascular disease risk is particularly concerning in light of projections from the Centers for Disease Control and Prevention (CDC), which estimate that 57% of children today will develop obesity by 35 years of age [3]. By compounding multiple traditional CVD risk factors such as insulin resistance, high blood pressure, and dyslipidemia, obesity exacerbates endothelial dysfunction and the CVD risk [4,5].

The link between obesity and CVD risk is further complicated by obesity-induced dysbiosis, which disrupts gut-derived metabolite profiles [6,7]. These imbalances are not yet recognized as contributing factors to CVD risk. One key consequence of obesity-induced dysbiosis is dysregulation in the metabolism of the essential amino acid tryptophan (Trp) [8]. Bacterial species such as *Bacteroides*, *Lactobacillus*, and *Bifidobacteria*—which express enzymes involved in the indole (i.e., exogenous) pathway of Trp metabolism [9,10]—are reduced in children with obesity. This microbial shift may contribute to metabolic dysregulation and CVD risk [8,11].

Emerging research underscores the role of Trp-derived metabolites in health and disease [12]. In rodents, for instance, a 12-week oral supplementation with indole-3-propionic acid (IPA) and indole-3-acetic acid (IAA) reduced diet-induced hepatic steatosis and inflammation [13]. Similarly, insulin resistance as assessed by the homeostatic model assessment for insulin resistance (HOMA-IR) was lower in rats fed an IPA-enriched diet compared to controls [14]. A 7-week IPA supplementation in a murine model of heart failure resulted in reduced weight gain, improved insulin sensitivity, decreased fasting cholesterol levels, and enhanced diastolic function [15]. Consistent with these experimental findings, the limited observational studies in humans have reported that adults with hepatic fibrosis and type 2 diabetes exhibit lower serum IPA levels compared to those without either condition [16].

Despite evidence in animals that gut-derived metabolites of the Trp pathway regulate inflammatory and metabolic processes, there is a notable scarcity of research examining their association with markers of vascular health in children and adults. Adolescence represents a critical window for investigating these mechanisms. This developmental stage is marked by puberty-induced insulin resistance and an unfavorable metabolic profile [17]. Additionally, obesity prevalence peaks during adolescence [18], making it a crucial time for studying emerging cardiovascular risk factors. To address this limitation, we conducted a secondary analysis of baseline samples collected in adolescents with obesity who participated in a randomized controlled trial (NCT04342390) [19]. The present study investigated the correlation between serum levels of gut-derived Trp metabolites and markers of cardiovascular health, including blood pressure, obesity severity, fasting lipid levels, endothelial function, markers of insulin resistance, and platelet health as measured by the spare respiratory capacity (SRC). Based on the published evidence, we hypothesized that serum levels of gut-derived Trp metabolites, specifically IPA and IAA, are associated with improved markers of cardiovascular and metabolic health, including blood pressure, fasting lipids, endothelial function, insulin sensitivity, and platelet health, in adolescents with obesity.

## 2. Materials and Methods

### 2.1. Subjects

Data from 28 adolescents enrolled in a randomized controlled trial (NCT04342390) at the Arkansas Children’s Nutrition Center and Arkansas Children’s Research Institute were included in the analysis. Participants had obesity and were 13 to 18 years old, from diverse racial and ethnic backgrounds, and in late puberty (Tanner stage IV or V). Adolescents with diabetes and an established diagnosis of metabolic dysfunction-associated steatosis liver disease (MASLD) were excluded. Additionally, those taking medications known to influence hepatic metabolism, such as metformin or steroids, were not eligible to participate. The study protocol was approved by the Institutional Review Board (IRB) at the University of Arkansas for Medical Sciences (IRB #260671). Written informed consent was obtained from the parents, and assent was provided by all participants under 18 years of age.

### 2.2. Anthropometry and Body Composition

Body weight and height were measured to the nearest 0.1 kg and 0.1 cm, respectively, using a digital scale (Seca 877, Seca GmbH & Co. KG, Hamburg, Germany). Measurements were taken in triplicate, and the average of the three values was used for analyses. Body mass index (kg/m^2^, BMI) and BMI percentiles were calculated using the CDC Child and Teen BMI Calculator [20]. Obesity was defined as a BMI at or above the 95th percentile for age and sex. Additionally, obesity severity was assessed using the percentage of the 95th percentile for BMI (%BMIp95). Class I obesity was defined as a BMI at or above the 95th percentile but less than 120% of the 95th percentile, or a BMI of 30 kg/m^2^ or greater, whichever value was lower. Class II obesity: BMI at or above 120% but less than 140% of the 95th percentile or a BMI of 35 kg/m^2^ or greater, whichever value was lower. Class III obesity: BMI at or above 140% of the 95th percentile or a BMI of 40 kg/m^2^ or greater, whichever value was lower [21]. Body fat mass (FM, kg) and lean body mass (LBM, kg) were measured using dual-energy x-ray absorptiometry (DXA, Horizon-A with Advanced Body Composition™, Hologic, Bedford, MA, USA). FMI index [FMI = FM (kg)/height (m^2^)] z-scores were computed using normative values in children [22].

### 2.3. Markers of Cardiometabolic, Vascular, and Platelet Health

#### 2.3.1. Blood Pressure

Systolic blood pressure (SBP, mmHg) and diastolic blood pressure (DBP, mmHg) were measured with an oscillometric electronic device using standardized techniques. Blood pressure status was defined according to the 2017 guidelines from the American Academy of Pediatrics [23]. Children with elevated blood pressure status or hypertension status were considered to have high blood pressure (HBP).

#### 2.3.2. Cardiorespiratory Fitness

Peak oxygen uptake (VO_2 peak_, mL/min) was measured using an incremental cycle ergometer stress test. The participants wore a heart rate monitor (Zephyr Bluetooth Wireless Heart Rate Sensor, Medtronic, Boulder, CO, USA) and an indirect calorimetry facemask. Oxygen uptake and carbon dioxide excretion (mL/min) were continuously measured during the test using a metabolic cart (Medgraphics Ultima PFX^®^ system, MGC Diagnostics Corporation, St. Paul, MN, USA). Exertion level was assessed via the OMNI scale [24]. VO_2 peak_ was deemed achieved if at least one of the following criteria was met: (1) rating of perceived exertion (RPE) of ≥8 on the OMNI scale, (2) peak heart rate of ≥185 beats per minute, and/or (3) respiratory exchange ratio (RER) ≥ 1.0. RER was defined as the ratio of the volume of carbon dioxide (CO_2_) released and the volume of oxygen (O_2_, mL/min) consumed. VO_2 peak_ was normalized to LBM and expressed in mL∙min^−1^∙kg^−1^ LBM.

#### 2.3.3. Blood Analytes

Blood was drawn following an overnight fast via venipuncture of the antecubital vein. Blood used for platelet isolation was collected without a tourniquet in an EDTA vacutainer, kept at room temperature, and processed within 30 min. Serum concentrations of glucose (mg/dL), triglyceride (TG, mg/dL), total cholesterol (TC, mg/dL), and low-density lipoprotein cholesterol (LDL-C, mg/dL) were measured using an RX Daytona clinical analyzer (Randox Laboratories-US Limited, Kearneysville, WV, USA). TG levels were classified as borderline if values were between 90 and 129 mg/dL and classified as high if values were ≥130 mg/dL. TC levels were considered borderline if between 170 and 190 mg/dL, and high if ≥200 mg/dL [24]. Insulin concentration (μIU/mL) was measured using an enzyme-linked immunosorbent assay (R&D Systems, Minneapolis, MN, USA). The homeostatic model assessment for insulin resistance (HOMA-IR) was calculated using the following equation:

HOMA-IR = [Fasting insulin (μIU/mL) × Fasting glucose (mg/dL)]/450

#### 2.3.4. Two-Hour Oral Glucose Tolerance Test

A standardized 2 h oral glucose tolerance test (OGTT) was performed. Serum glucose and insulin levels were measured at 0 and 120 min. Participants consumed a standard OGTT glucose drink (1.75 g/kg, up to a maximum of 75 g) within 5 min following blood collection at time 0. Impaired fasting glucose was defined as glucose > 100 mg/dL at time 0, and impaired glucose tolerance as glucose ≥ 140 but <200 mg/dL at 120 min.

#### 2.3.5. Platelet Mitochondria Respiration by High-Resolution Respirometry

Whole blood was centrifuged at 200× *g* for 10 min (acceleration 9, no brakes) at room temperature to separate platelet-rich plasma. EGTA (100 mM starting concentration) was added (10% vol/vol) to the platelet-rich plasma, which was then centrifuged at 1000× *g* for 10 min at room temperature. Platelets were washed with phosphate-buffered saline (PBS) containing 10 mM EGTA and centrifuged at 1000× *g* for 5 min at room temperature. Platelets were resuspended in a final volume of 500 µL of EGTA-supplemented PBS and counted as previously described [25]. Platelet Spare Respiratory capacity (SRC) was used as an indicator of platelet function and was calculated as the difference between maximal respiration and routine respiration. Routine respiration (pmol O_2_·s^−1^·10^−6^ cells) was measured before permeabilization of the platelet membrane. Maximal respiration was measured following the addition of the protonophore carbonyl cyanide m-chlorophenyl hydrazone (CCCP). Details on the substrate-uncoupler-inhibitor titration (SUIT) protocol used in the present study can be found at https://www.bioblast.at/index.php/SUIT-002_O2_ce-pce_D007a (accessed on 16 January 2025).

#### 2.3.6. Intrahepatic Triglyceride Content

Intrahepatic triglyceride content was quantified using a 1.5 T magnetic resonance imaging (MRI) scanner (Philips Healthcare, Best, The Netherlands) at Arkansas Children’s Hospital. In brief, a multi-echo, multi-slice gradient-echo pulse sequence was used to acquire in-phase and out-of-phase images of the entire liver during a breath-hold, using a repetition time (TR) of 150 ms, a flip angle of 25 degrees, and echo times of 2.3 ms, 4.6 ms, and 9.2 ms. A triple-echo method was employed to control for the confounding effects of intrinsic T2 and T1 relaxation. Raw MRI images were transferred to a workstation equipped with MATLAB software, version ML 2018b (The MathWorks Inc., Natick, MA, USA), where customized scripts were used for data analysis. Two raters, blinded to participant data, delineated a region of interest (ROI) for each subject that encompassed as much of the liver parenchyma as possible while excluding intrahepatic vessels, perihepatic fat, and liver margins. The mean signal intensity within each ROI was calculated across all three echo times, and liver fat content was derived from these signal intensities [26]. MASLD was defined as a liver fat percentage greater than or equal to 5% [27].

#### 2.3.7. Flow Mediated Dilation

Endothelial function was measured using ultrasound (GE Vivid 7 Ultrasound system with 5.0–13 MHz linear transducer, GE Healthcare, Chicago, IL, USA) to assess brachial artery flow-mediated dilation (FMD). Three electrocardiogram (ECG) electrodes were placed on the torso of the subject to capture ultrasound measurements at identical times during the cardiac cycle (i.e., ECG-gating of ultrasound images). A blood pressure cuff was placed around the lower arm, and it was inflated for 5 min, followed by rapid deflation of the cuff. The diameter of the brachial artery was measured using edge detection software (Vascular Tool, Medical Imaging Applications, Coralville, IA, USA) from ultrasound images captured during the R wave of the cardiac cycle (Via ECG-gating). The software was calibrated to display a distance scale of 10 mm. Baseline measurements were analyzed with a region of interest (ROI) with a width range of 2.5–3 mm, while the height was adjusted to ensure the whole artery could be captured. Following the baseline analysis, deflation measurements were performed. Quality control was conducted with a confidence threshold of 50%. The calculation of FMD is presented as a percentage of change in the vessel caliber.

### 2.4. Indole Metabolites Analysis

#### 2.4.1. Materials and Reagents

Indole-3-propionic acid (IPA), indole, indole-3-aldehyde (IAld), indole-acrylic acid (IA), and indole-3-acetaldehyde (IAAld) were purchased from Sigma Aldrich (ST. Louis, MO, USA). Indole-3-lactic acid (ILA) and indole-3-acetic acid (IAA) were obtained from Combi-Blocks, Inc. (San Diego, CA, USA), while indoxyl sulfate (IS) was sourced from Toronto Research Chemicals (Toronto, ON, Canada). All isotopically labeled compounds used as internal standards were obtained from Cambridge Isotope Laboratories, Inc. (Tewksbury, MA, USA). Reagents used were of mass spectrometry grade and were supplied by Fisher Scientific (Pittsburgh, PA, USA).

#### 2.4.2. Quantification of Tryptophan Metabolites

Indole metabolites from plasma were quantified using a validated ultra-performance liquid chromatography-tandem mass spectrometry (UPLC-MS/MS) assay. A standard mixture was prepared by combining all stock standards (1 mg/mL), which were dissolved in either ethanol or water according to the manufacturer’s instructions. This mixture was used to perform a 15-point reference standard calibration ranging from 0 to 10 µg/mL. A mixture of internal standards (ISTD) was also prepared and spiked into every sample and calibration standard. Plasma samples (100 µL) were extracted with 500 µL of 80% methanol containing ISTD (250 ng/mL), mixed at 4 °C and 2000 rpm for 30 min, and centrifuged at 4 °C at 18,000× *g* for 10 min. A volume of 250 µL of the supernatant was transferred to a 96-well plate and evaporated to dryness using a SpeedVac. Dried extracts were reconstituted in 40 µL of 50% methanol. The concentration of each targeted metabolite was determined based on an optimized calibration curve and normalized using the ISTD. A pooled quality control (QC) sample was prepared by combining aliquots from all sample extracts and injected every 12 samples to assess assay reproducibility and monitor batch effects.

Chromatographic separation was performed using a Water ACQUITY Premier UPLC system, and quantification was carried out on a Xevo TQ-S micro triple quadrupole mass spectrometer (Waters Corporation, Milford, MA, USA). Separation was achieved using an ACQUITY Premier UPLC fitted with an HSS T3 C18 reverse-phase column (2.1 × 100 mm, 1.8 µm), maintained at 50 °C. Samples were kept at 10 °C prior to injection. The flow rate was set at 500 µL/min with an injection volume of 4 µL. The mobile phases consisted of 0.1% formic acid in 10 mM ammonium formate (solvent A) and 0.1% formic acid in methanol (solvent B). The 12 min elution gradient was as follows: hold at 0% B for 1 min, ramp to 30% B over 4 min, increase to 100% B over the next 4 min, hold at 100% B for 2 min, return to 0% B in 0.1 min, and hold at 0% B until 12 min.

Quantification was performed on a Xevo TQ-S Micro triple quadrupole mass spectrometer. Data acquisition and analysis were conducted using MassLynx software (V4.2 SCN1035). Multiple reaction monitoring (MRM) data were acquired in both positive and negative electrospray ionization modes. The assay demonstrated linear standard calibration curves up to 10,000 ng/mL, with limits of quantitation ranging from 2.4 to 29.6 ng/mL. These characteristics support the assay’s suitability for measuring a wide dynamic range of metabolite concentrations in biological samples, such as the vaginal secretions and serum used in this study.

### 2.5. Dietary Intake and Medical History Questionnaire

Dietary intake was assessed using the Screener Food Frequency Questionnaire (FFQ) designed for children aged 2–17 years. The screener, which asks participants to recall food consumption over the past week, was administered by a trained research staff member. To improve the accuracy of the portion size report, measurement cups and visual aids (e.g., pictures of plates and portion sizes) were provided during the interview.

Medical history was assessed using a structured questionnaire designed to capture information on existing medical conditions, use of medications, dietary supplement intake, and levels and frequency of physical activity. Specifically, the questionnaire documented whether children engaged in regular exercise, the type of activity performed, and the number of days per week they participated.

### 2.6. Statistical Analysis

Continuous variables measured on an interval scale are summarized as mean ± standard deviation (SD), while categorical variables on an ordinal or nominal scale are presented as frequencies and percentages. Mean comparisons between boys and girls were performed using the Mann–Whitney U test. Spearman’s rank correlation coefficient was used to evaluate correlations between serum indole metabolite concentrations and variables of interest. When potential confounders were identified, generalized linear regression models were employed to estimate adjusted values, and Spearman correlations were subsequently computed using these adjusted values. All statistical analyses were conducted using SAS^®^ 9.4 (Cary, NC, USA).

## 3. Results

### 3.1. Subject Characteristics

The participants had a mean age of 15.5 ± 1.4 years and a BMI of 36 ± 6.4 kg/m^2^. Twelve children (43%) had class I obesity, 7 (25%) class II obesity, and 9 (32%) class III obesity. The sample comprised 54% boys and 46% girls, with no significant difference in gender distribution (*p* = 0.7055). Half of the participants were Hispanic-White (HW), 39% (n = 11) were non-Hispanic Black (NHB), and 11% (n = 3) were non-Hispanic White (NHW) (Table 1).

### 3.2. Correlation of Dietary Intake, Physical Activity, and Use of Medications and Supplements with Serum Concentrations of Gut-Derived Tryptophan Metabolites

The participants had a mean intake of 1345.58 ± 825.15 kilocalories per day, which included 58.42 ± 33.94 gr of proteins, 153.98 ± 108.36 gr of carbohydrates, and 57.14 ± 34.02 gr of fat. There was no association between kilocalorie or macronutrient intake and serum levels of indole metabolites. When medication use was assessed, 39% of adolescents (n = 11) reported taking at least one medication. The reported medications included antihypertensives (n = 1), antihistamines and asthma-related treatments (n = 5), antidepressants or anxiolytics (n = 2), migraine medications (n = 2), and medications for other conditions (n = 1). No participants reported using antibiotics, probiotics, or prebiotics.

Fifty-four percent of adolescents (n = 15) reported taking either multivitamins or vitamin D supplements. No associations were observed between supplement use and levels of indole metabolites.

### 3.3. Metabolic Profile of Adolescents

The average liver fat percentage among subjects was 6.0 ± 4.3%, with a range of 1.1% to 18.6%. MASLD was identified in 46% (n = 13) of participants, of whom 71% (n = 10) were HW. HBP was present in 57% (n = 16) of the participants. Combined MASLD and HBP were observed in 27% (n = 8) of the cohort, with 7 out of 8 individuals being HW. Additionally, 25% (n = 7) of the participants exhibited impaired fasting glucose, elevated fasting serum triglyceride (TG) levels, and borderline or high total cholesterol (TC). Impaired glucose tolerance, as determined by the oral glucose tolerance test (OGTT), was present in 21% (n = 6) of the adolescents (Table 1).

### 3.4. Correlation Between Subject Characteristics and Serum Levels of Gut-Derived Trp Metabolites (Table 2)

Three out of the eight measured indole metabolites showed positive correlations with male sex: IA (rho = 0.43, *p* = 0.0240), ILA (rho = 0.64, *p* = 0.0002), and IS (rho = 0.38, *p* = 0.0481). Mean comparisons between boys and girls are presented in Table 3.

**Table 2:** Spearman correlation coefficients between indole metabolites and participant characteristics.

Variable	IAAld	IA	IAld	ILA	IAA	IPA	IS	Indole
	rho	rho	rho	rho	rho	rho	rho	rho
Age	−0.50 **	−0.19	0.07	−0.12	−0.26	−0.09	0.04	0.04
Male sex	0.21	0.43 *	0.28	0.64 ***	0.14	0.18	**0.38**	0.11
Non-Hispanic ethnicity	−0.15	−0.10	−0.35	0.03	−0.41 *	−0.19	−0.34	−0.46 **
FMIZ	−0.09	−0.25	−0.15	−0.16	−0.25	−0.30	−0.31	−0.40 *
BMIp95	0.17	−0.10	−0.13	−0.29	−0.22	−0.43 *	−0.14	−0.41 *
SBP	0.24	0.04	0.03	0.18	−0.10	−0.17	0.05	0.12
DBP	0.17	−0.27	−0.15	−0.25	0.09	−0.36	−0.15	−0.02
Fasting glucose	0.14	0.30	0.04	0.02	−0.11	−0.02	0.47 **	−0.24
2 h glucose	0.45 *	0.03	0.13	0.07	−0.08	−0.17	0.19	−0.28
HOMA-IR	−0.05	−0.24	0.30	−0.10	−0.02	−0.16	0.33	−0.12
VO_2 peak_	0.18	0.25	0.36 *	0.24	0.64 ***	0.29	−0.01	0.34
IHTG	0.13	0.07	0.38 *	0.04	0.43 *	0.02	0.23	0.26
TC	−0.30	−0.35	−0.64 ***	−0.46 *	−0.57 **	−0.36 *	−0.20	−0.34
TG	0.06	−0.22	0.18	−0.04	0.00	−0.03	0.03	0.03
LDL-C	−0.29	−0.33	−0.62 ***	−0.58 **	−0.53 **	−0.39 *	−0.30	−0.28
Leptin	−0.14	−0.31	−0.18	−0.44 *	−0.22	−0.28	−0.36	−0.24
Adiponectin	0.06	−0.07	**0.13**	0.13	0.08	0.08	−0.06	0.30
Platelet SRC	0.14	0.32	0.28	0.46 *	0.20	0.10	0.53 **	−0.03
FMD	0.23	0.29	−0.38 *	0.17	−0.10	−0.05	−0.11	−0.34

IAAld = indole-3-acetaldehyde; IA = Indole-acrylic acid; IAld = Indole-3-aldehyde; ILA = Indole-3-lactic acid; IAA = Indole-3-acetic acid; IPA = Indole-3-propionic acid; IS = Indoxyl sulfate; FMIZ = fat mass index z-score; BMIp95 = body mass index at 95 percentile; HOMA-IR = homeostatic model assessment of insulin resistance; VO_2 peak_ = peak oxygen uptake; SBP = systolic blood pressure; DBP = diastolic blood pressure; IHTG = intrahepatic triglyceride; TC = total cholesterol; TG = triglyceride; LDL-C = low-density lipoprotein cholesterol; SRC = spare respiratory capacity; FMD = flow mediated dilation. * *p* < 0.05; ** *p* < 0.01; *** *p* < 0.001. Note: Bolded values indicate statistical significance.

**Table 3:** Serum concentration of indole metabolites by sex.

Tryptophan Metabolites (ng/mL)	n = 28 Total	n = 15 Boys	n = 13 Girls	*p* Value
IAAld	6.3 ± 5.9	7.9 ± 7.4	4.9 ± 4.1	0.2788
IAA	493.3 ± 347.4	475.0 ± 195.0	509.2 ± 446.8	0.4752
IA	11.7 ± 6.9	14.6 ± 8.5	9.1 ± 4.1	**0.0270**
IAld	2.1 ± 0.8	2.4 ± 1.0	1.8 ± 0.5	0.1464
ILA	187.0 ± 48.1	218.4 ± 39.2	159.79 ± 37.83	**0.0008**
IPA	227.2 ± 136.1	251.9 ± 139.6	205.70 ± 134.04	0.3450
IS	1713.2 ± 805.1	2053.3 ± 876.5	1418.36 ± 625.33	0.0503
Indole	1.9 ± 1.0	2.1 ± 1.2	1.81 ± 0.63	0.5961

Data presented in means and standard deviation. IAAld = indole-3-acetaldehyde; IAA = Indole-3-acetic acid; IA = Indole-acrylic acid; IAld = Indole-3-aldehyde; ILA = Indole-3-lactic acid; IPA = Indole-3-propionic acid; IS = Indoxyl sulfate. Note: Bolded values indicate statistical significance.

**Figure 1:**
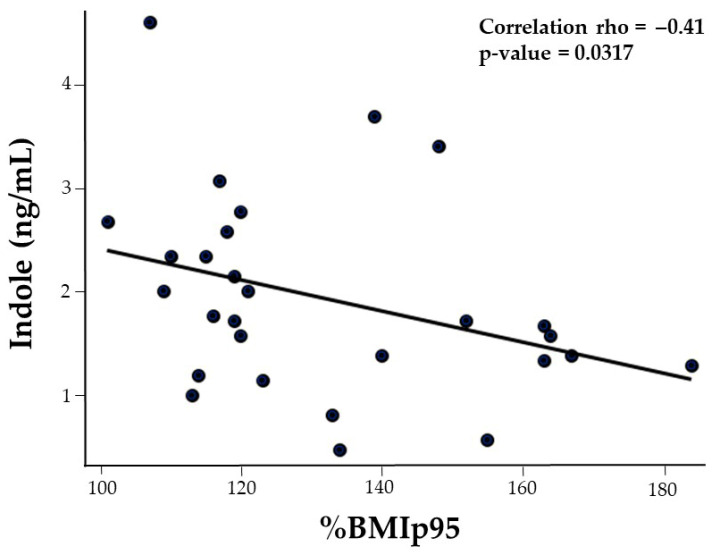
Scatterplot illustrating the negative correlation between serum indole concentrations and %BMIp95 in adolescents with obesity (black circles).

Ethnicity differentially correlated with IAA and indole serum concentrations (Table 2). Mean comparisons showed that non-Hispanic adolescents had lower serum IAA (Non-Hispanic: 474.1 ± 466.2 ng/mL vs. Hispanic: 512.5 ± 180.6 ng/mL, *p* = 0.0346) and indole (non-Hispanic: 1.5 ± 0.7 ng/mL vs. Hispanic: 2.9 ± 0.1 ng/mL, *p* = 0.0179) serum concentrations. IPA (rho = −0.43, *p* = 0.213) and indole (rho = −0.41, *p* = 0.0317) were negatively associated with BMI expressed as a percentage of the 95th percentile (%BMIp95) (Figure 1), while age inversely correlated with IAAld (rho = −0.50, *p* = 0.0072).

### 3.5. Correlation of Cardiometabolic, Vascular, and Platelet Health Markers with Gut-Derived Trp Metabolites (Table 2)

#### 3.5.1. Diastolic Blood Pressure 

**Figure 2:**
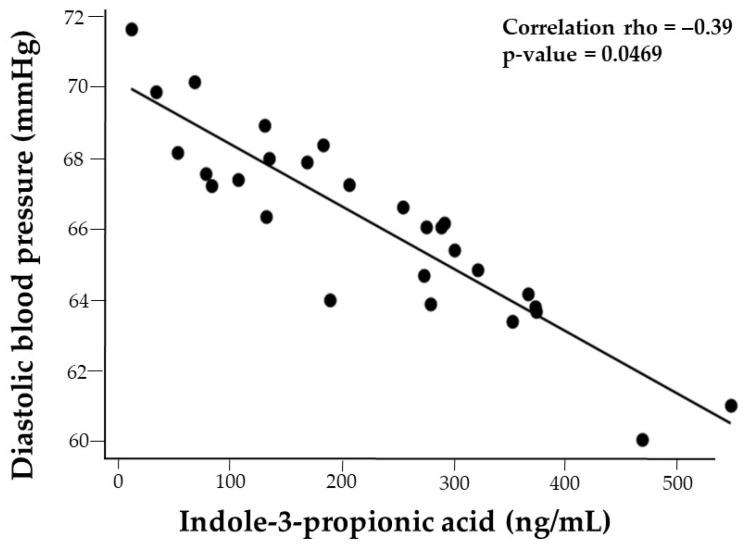
Adjusted correlation plot illustrating the association between indole propionic acid (IPA) and diastolic blood pressure (DBP) in adolescents with obesity. The adjusted plot accounts for obesity status expressed as %BMIp95.

There was a marginal correlation between DBP and IPA (*p* = −0.36, *p* = 0.0609). Given that IPA was negatively correlated with the %BMIp95, the association between IPA and DBP was adjusted for %BMIp95, resulting in a correlation of rho = −0.39, *p* = 0.0469 (Figure 2).

#### 3.5.2. Fasting Serum Glucose and Oral Glucose Tolerance Test

**Figure 3:**
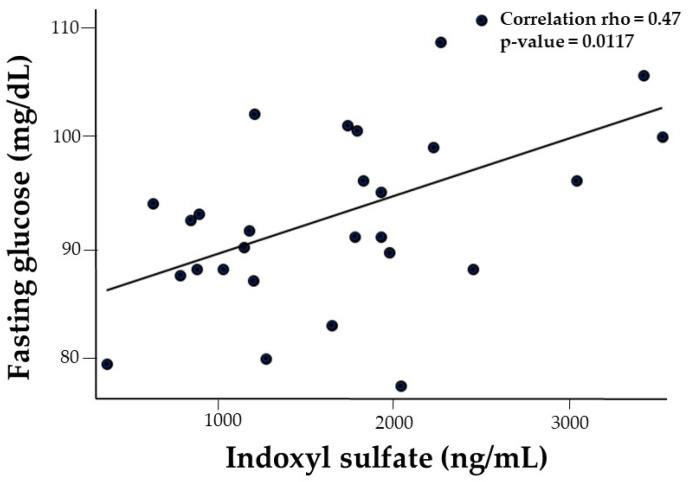
Unadjusted correlation between indoxyl sulfate (IS) and fasting glucose in adolescents with obesity.

There was a positive correlation between IS and fasting serum glucose concentration (rho = 0.47, *p* = 0.0117). This association remained significant after adjusting for sex (Figure 3)

The OGTT 2 hr glucose concentration directly correlated with IAAld (rho = 0.45, *p* = 0.0150). This association remained significant after controlling for age (rho = 0.42, *p* = 0.0265).

#### 3.5.3. Fasting Serum Lipids

**Figure 4:**
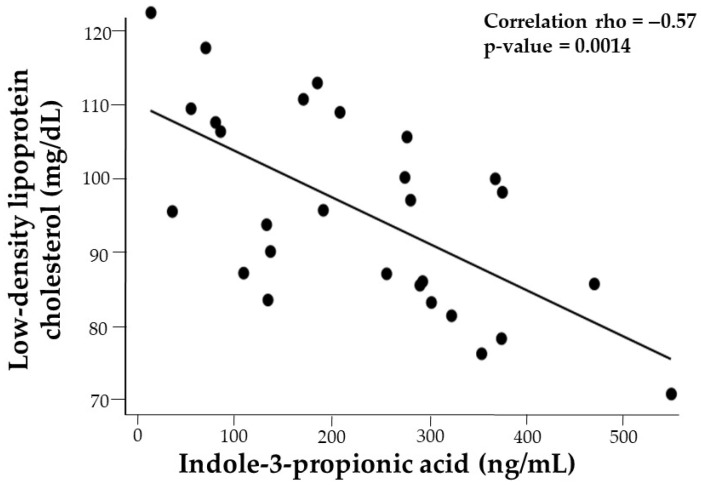
Sex and %BMIp95 adjusted correlation plots illustrating the association between indole-3-propionic acid (IPA) and low-density lipoprotein cholesterol.

TC negatively correlated with IAld (*p* = −0.64, *p* = 0.0003), ILA (*p* = −0.46, *p* = 0.0131), and IPA (*p* = −0.36, *p* = 0.0583). Similarly, LDL-C showed negative correlations with IAld (*p* = −0.62, *p* = 0.0004), IAA (*p* = −0.53, *p* = 0.0041), and IPA (*p* = −0.39, *p* = 0.0424). After adjusting for ethnicity and sex, the correlations of ILA and IAld with plasma lipids were no longer significant. However, the correlation of IPA with LDL-C (rho = −0.57, *p* = 0.0014) and TC (−0.55, *p* = 0.002) gained significance after controlling for %BMIp95 and sex (Figure 4).

#### 3.5.4. Adipocytokines

Serum leptin concentrations were negatively correlated with ILA (rho = −0.45, *p* = 0.0174); however, this association was no longer significant after adjusting for sex. No significant associations were observed between indole metabolites and adiponectin (Table 2).

#### 3.5.5. Platelet Function: Spare Respiratory Capacity (SRC)

SRC positively correlated with ILA (rho = 0.46, *p* = 0.0246) and IS (rho = 0.53, *p* = 0.0082). However, these correlations were no longer significant after correcting for %BMIp95 and sex, respectively.

#### 3.5.6. Cardiorespiratory Capacity (VO_2 peak_)

VO_2 peak_ positively correlated with IAld (rho = 0.36, *p* = 0.0597) and IAA (rho = 0.64, *p* = 0.0002). These correlations were no longer significant after correcting for %BMIp95 and sex.

#### 3.5.7. Flow-Mediated Dilation

No significant correlations were observed between FMD and gut-derived Trp metabolites.

## 4. Discussion

The purpose of this study was to evaluate the association of gut-derived Trp metabolites with markers of cardiometabolic health and platelet health measured by the spare respiratory capacity. The main findings from this study were that gut-derived tryptophan metabolites were associated with age, sex, ethnicity, and obesity severity (i.e., %BMIp95) in adolescents with obesity. Also, diastolic blood pressure and plasma lipids (i.e., total cholesterol and LDL-C) negatively correlated with indole-3-propionic acid even after considering important confounders. Finally, indoxyl sulfate, a uremic toxin that increases CVD risk in patients with chronic kidney disease, positively correlated with fasting plasma glucose. No significant associations were observed between gut-derived tryptophan metabolites and endothelial function, cardiorespiratory fitness, or platelet bioenergetics.

We observed a negative correlation between indole-3-acetaldehyde serum levels and age. Human studies examining these relationships are scarce, limiting our understanding of how developmental processes influence the tryptophan metabolic pathway in youth. However, evidence from animal models provides important insights. A study conducted in male mice examined the effects of chronological age and gut microbiota composition on the metabolome at four developmental stages (3, 6, 18, and 28 months) [28]. The authors reported negative correlations between age and the levels of gut-derived tryptophan metabolites, specifically indole (rho = −0.82, *p* = 0.002) and indole-3-lactic acid (rho = −0.68, *p* = 0.020). Notably, changes in the metabolome paralleled changes in microbiome composition, suggesting that shifts in microbial communities may contribute to age-related differences in the metabolomic profile. In our study, only indole-3-acetaldehyde was inversely associated with age. Unlike animal models, however, human studies face challenges in disentangling the effects of aging from confounding lifestyle factors such as diet, medications, and comorbidities. Although our study did not include microbiome analysis, the findings, taken together with existing literature, underscore the importance of considering microbial dynamics in age-related health outcomes and highlight the need to identify critical windows for tailored intervention strategies.

Another finding from this study was that indole-3-acrylic acid, indole-3-lactic acid, and indoxyl sulfate were present in higher concentrations in boys compared to girls. Although no studies have specifically focused on sex-based differences in gut-derived tryptophan metabolites, there is substantial evidence demonstrating sexual dimorphism in gut microbiome composition, which may contribute to differences in metabolite profiles [29]. A large case–control study (n = 2114; mean age 68.7 years) performed targeted metabolomics profiling of 357 metabolites and identified sex-associated metabolites linked to incident stroke, coronary heart disease, hypertension, and chronic kidney disease [30]. In agreement with our study, indole-3-lactic acid was higher in men compared to women. Furthermore, the authors reported a positive association between indole-3-lactic acid and the odds of chronic kidney disease (OR = 3.01, CI = 2.27–3.98, *p* < 0.0001). Taken together, our findings support the importance of considering sex as a biological variable in the gut metabolism of tryptophan. Whether the sex differences observed herein are associated with health outcomes or influence the health trajectories of adolescents warrants further investigation.

A novel finding of this study was the association between Hispanic ethnicity and higher circulating levels of indole-3-acetic acid and indole, with Hispanic participants exhibiting greater concentrations of these metabolites compared to non-Hispanic individuals. While studies specifically examining the relationship between ethnicity and serum levels of indole metabolites are limited, traditional Hispanic diets are generally characterized by higher intakes of vegetables, fruits, seafood, plant-based proteins, and dietary fiber [31]. These dietary patterns are known to influence both the diversity and relative abundance of the gut microbiome [32], and consequently, the production of gut-derived metabolites. Prior studies have also demonstrated a strong relationship between dietary fiber intake and indole metabolite levels [33,34], highlighting the critical role of gut microbiome composition in tryptophan metabolism. In our study, we did not observe a significant association between total caloric or macronutrient intake and serum indole metabolites. However, dietary intake was assessed using a brief dietary screener rather than comprehensive food records, which may have limited the accuracy of intake estimates. Additionally, dietary acculturation may help explain the lack of differences in dietary composition observed between ethnic groups in this study. Finally, although genetic polymorphisms related to indole-3-propionic acid have been reported in individuals at high risk for esophageal cancer, ethnic differences in these polymorphisms have not yet been documented [35].

We also observed that indole-3-propionic acid levels inversely correlated with total cholesterol, LDL-C, and diastolic blood pressure. These associations remained significant after adjusting for BMI and sex, suggesting a potential protective role of indole-3-propionic acid in cardiovascular health during adolescence. This observation aligns with findings from a porcine model investigating the effects of inulin, a fermentable prebiotic fiber, on microbiota and gut-derived metabolites [36]. The inulin-fed group (n = 6) had lower total cholesterol, and indole-3-propionic acid was inversely correlated with LDL-C (rho = −0.59, *p* = 0.04). Findings from a prospective study (n = 11,972) showed that higher levels of indole-3-propionic acid were associated with a lower incidence of ischemic heart disease and peripheral artery disease [37]. These results are consistent with those of a cross-sectional study (n = 100) in which the probability of advanced atherosclerotic disease decreased with increasing plasma concentrations of indole-3-propionic acid (OR = 0.27, 95% CI: 0.09–0.91; *p* = 0.02) [38]. Mechanistically, indole-3-propionic acid has been shown to promote cholesterol efflux from macrophages via the ATP-binding cassette transporter A1 (miR-142-5p/ABCA1) signaling pathway. Notably, dysregulation of this pathway, along with lower circulating levels of indole-3-propionic acid, has been observed in patients with coronary artery disease [39]. However, to date, there is a lack of research examining these associations in children. The present study may therefore represent an important early contribution to understanding the potential role of indole-3-propionic acid in pediatric cardiovascular health.

Another important observation from this study was the direct association between indoxyl sulfate and fasting serum glucose levels in adolescents. This finding aligns with a preclinical study by Opdebeeck et al. [40], which found that chronic exposure to indoxyl sulfate in a rat model of chronic kidney disease led to the formation of vascular calcifications and elevated fasting glucose levels. Using proteomics analysis, the authors reported activation of inflammatory and coagulation pathways, along with reduced expression of the insulin receptor and peroxisome proliferator-activated receptor. Taken together with our findings, this evidence suggests that indoxyl sulfate may be associated with disruptions in glucose homeostasis. In the present study, seven adolescents had impaired fasting glucose, and their indoxyl sulfate levels were 1.6 times higher compared to those without impaired fasting glucose. This supports a potential link between indoxyl sulfate and early disturbances in glucose metabolism, indicating that this uremic toxin may contribute to metabolic dysfunction beyond its known role in kidney disease. Further studies are warranted to elucidate the underlying mechanisms and clinical implications in at-risk pediatric populations.

This study is limited by its small sample size and the absence of microbiome analysis. Nonetheless, it is strengthened by the inclusion of adolescents with obesity, a population at elevated risk for cardiometabolic disease, and by the comprehensive profiling of indole pathway metabolites using advanced analytical techniques. Participants also underwent in-depth metabolic phenotyping, including liver fat quantification by magnetic resonance imaging, along with assessments of flow-mediated dilation, fitness testing, and platelet bioenergetics, which enhance the scientific rigor and innovation of the study.

## 5. Conclusions

In conclusion, this study provides new insight into the complex relationships between gut-derived tryptophan metabolites and cardiometabolic health in adolescents with obesity. We observed that individual metabolite profiles varied by age, sex, ethnicity, and obesity severity, with specific metabolites such as indole-3-acetaldehyde and indoxyl sulfate showing distinct associations with age and fasting glucose, respectively. Our findings support the notion that microbial metabolism of tryptophan is associated with early metabolic disturbances, including dyslipidemia and impaired glucose regulation in adolescents with obesity.

## Figures and Tables

**Table 1:** Participant characteristics.

Variables	n = 28	Boys n = 13	Girls n = 15	*p* Value
Age, (years)	15.5 ± 1.4	15.7 ± 1.5	15.4 ± 1.4	0.6954
BMI, (kg/m^2^)	36 ± 6.4	34.2 ± 5.8	38.8 ± 6.4	0.0503
BMI Category, n (%)				**0.0459**
Class I	12 (43)	8 (62)	4 (27)	0.0678
Class II	7 (25)	3 (23)	4 (27)	**0.0462**
Class III	9 (32)	2 (15)	7 (46)	0.0826
FMIZ	1.78 ± 0.38	1.6 ± 0.38	1.9 ± 0.4	0.1532
Race—Ethnicity, n (%)				
HW	14 (50)	8 (62)	6 (40)	
NHW	3 (11)	3 (23)	0 (0)	
NHB	11 (39)	2 (15)	9 (60)	
Liver fat, (%)	6 ± 4.30 (Range: 1.10–18.56)	7 ± 3.46	5 ± 4.98	0.1020
MASLD, n (%)	13 (46)	8 (61)	5 (33)	0.1617
HW	10 (71)	6 (46)	4 (27)	
NHW	1 (7)	0 (0)	0 (0)	
NHB	2 (18)	2 (15)	1 (6)	
HBP, n (%)	16 (57)	7 (54)	9 (60)	0.7700
HW	9 (53)	5 (38)	4 (27)	
NHW	1 (6)	1 (8)	0 (0)	
NHB	6 (55)	1(8)	5 (33)	
HBP and NAFLD, n (%)	8 (29)	4 (31)	4 (27)	0.8139
HW	7 (41)	4 (31)	3 (21)	
NHW	0 (0)	0 (0)	0 (0)	
NHB	1 (9)	0 (0)	1 (6)	
Systolic blood pressure, (mmHg)	121 ± 7	120 ± 6	121 ± 8	0.8355
Diastolic blood pressure, (mmHg)	66 ± 7	63 ± 8	69 ± 7	**0.0283**
Fasting glucose, (mg/dL)	93 ± 8	97 ± 7	90 ± 8	**0.0339**
Impaired fasting glucose, n (%)	7 (25)	5 (38)	2 (13)	
OGTT, 2 hr serum glucose, (mg/dL)	121 ± 30	116 ± 29	112 ± 27	0.1970
Impaired glucose tolerance, n (%)	6 (21)	6 (21)	6 (21)	0.1326
HOMA-IR	8.95 ± 6.34	8.95 ± 6.34	8.95 ± 6.34	0.8719
Total cholesterol, (mg/dL)	150 ± 27	137 ± 20	162 ± 28	**0.0270**
LDL-C, (mg/dL)	96 ± 21	85 ± 14	105 ± 21	**0.0057**
Triglycerides, (mg/dL)	111 ± 47	107 ± 34	114 ± 57	0.9449
VO_2 peak_ (ml∙min^−1^∙kg^−1^ LBM)	40 ± 7.01	43 ± 7.8	38 ± 5.4	0.0503
Physical activity, n (%)				**0.0060**
No exercise	13 (46)	3 (23)	10 (67)	**0.0235**
<3 days/week	10 (36)	5 (38)	5 (33)	0.7815
≥3 days/week	5 (18)	5 (38)	0 (0)	**0.0093**

Data presented as mean ± SD, counts, and percentages. BMI = body mass index; FMIZ = fat mass index z-score; HW = Hispanic white; NHW = non-Hispanic white; NHB = non-Hispanic Black; MASLD = metabolic dysfunction-associated steatotic liver disease; HBP = high blood pressure; OGTT = oral glucose tolerance test; HOMA-IR = homeostatic model assessment of insulin resistance; LDL-C = low-density lipoprotein cholesterol; VO_2 peak_= peak oxygen uptake; LBM = lean body mass Note: Bolded values indicate statistical significance.

## Data Availability

The raw data supporting the conclusions of this article will be made available by the authors upon request due to privacy restrictions.
